# Modern Anthropology

**Published:** 1893-12-30

**Authors:** 


### The Study of Man.

### III.—MODERN ANTHROPOLOGY.

Down to the beginning of the present century the development of anthropology, as sketched in a recent article, was simple and straightforward; and, in particular, those controversies had been fortunately avoided which lead to no permanent result, and tend to divert the attention of students from the main problems of the science. Its history, on the contrary, for the next sixty years is closely bound up, on more than one side, with that of a dispute which is really more a question of words and ideas, than of the facts to be interpreted by them. Linnæus, it is true, had divided his genus *Homo* into the three species *sapiens, ferus*, and *monstruosus*, but it is clear enough that his distinction was on the one hand almost mythical, and, on the other, really psychological and social; and in spite of the evident tendencies of the "école des idées" in biology, headed by Lamarck and Geoffroy St. Hilaire, the unity and stability of the human species remained practically undisputed until the close of the eighteenth century.

In 1801, however, Virey, using Camper's new craniological method, took up, in an otherwise worthless book, the position, that mankind was to be divided into two natural kinds, distinguishable by the amount of the facial angle. Virey's views are found cautiously adopted in England in a lecture by Lawrence in 1817, and seem to have obtained wide acceptance abroad; but no direct answer seems to have been made before Lacépèdés restatement of the old "monogenist" view in 1821. Virey was reinforced shortly afterwards by Bory de Saint Vincent with fifteen species (1825), supported by Pickering; and almost simultaneously by Desmoulins, first with eleven, afterwards (1826) with sixteen. Prichard and the English inquirers in general held to the older and simpler taxonomy, in agreement with Cuvier and the majority of his so-called "classical" school; while the American anthropologists Morton (1847), Nott (1854), and Gliddon (1857), together with D'Orbigny (l'Homme Americain, 1839) and Agassiz (1854-7) are the most extreme exponents of the "polygenistic" theory: Agassiz even contemplating the probability of an infinite number of species, distinguished by the smallest observable divergencies. It became gradually very clear that what was really in dispute was the very definition of species and of race, not the specific or racial value of this or that individual feature.

Two causes contributed to give this controversy an exaggerated importance. On the one hand the unity or plurality of the human species had become a crucial instance in the increasingly pressing general question of the characteristics of real kinds, of the permanence or variability of species; and, in detail, of the more and more perplexing phenomena of hybridity in plants and animals; a subject which Morton and Agassiz in particular had studied with great care, and whose views on which wholly determined their attitude with regard to specific differences in man.

The other cause was a social, and even a political one. When the equality and fraternity of man had become, partly through speculative agencies, the watchword of a practical creed, it was as important for the defence to find evidence to support its contention of the natural *in*equality of man, as for the attack to have a show of scientific argument on its side. The first London Anthropological Society, for example, founded in 1838, was a philanthropic institution pledged to examine the grounds of the so-called "natural slavery," advanced mainly by polygenistic writers; for if a negro was of another species than his master, what was to save him from the treatment appropriate to the horse or dog species? The inaugural discourse of the founder of the Paris Ethnological Society in the following year treats of a kindred subject: "Sur les Caractères Physicales des Races Humaines, Considerées dans leurs Rapports avec l'Histoire." And it has been seen that it was in America, where the slave question was most urgent, that the advocates of polygenism carried their arguments to the furthest extent.

The publication, however, of the "Origin of Species," followed so nearly by the Abolitionist crisis in the United States, marks a point, beyond which the whole controversy is seen to have lost its meaning, though it will be found discussed with spirit in so recent a book as De Quatrefages' "Histoire Générale des Races Humaines" (Paris, 1887-9). From 1859 onwards, in fact, and still more since the appearance of the "Descent of Man," in 1874, the problem is not so much "Are the white and the black races distinct?" as "How are they related to one another and to their common ancestor, and how have they come to occupy the geographical and biological positions which they respectively hold?"

A vast amount of useful work, however, was being done meanwhile in this period, and that in very various directions. As the field widened it became more and more impossible for individuals to have first-hand knowledge of all its sections; and, though the Société des Observateurs de l'Homme ceased to exist before the end of the great war, and neither the London Anthropological nor the Paris Ethnological Society outlived its immediate object, the foundation, in 1844, of the London Ethnological Society (the forerunner of the Anthropological Institute of 1863), and of the Société d'Anthropologie in Paris in 1859, testifies to the feeling that some medium of co-operation and interchange of ideas was wanted among so large a body of workers in such very different spheres; while within a very few years similar centres were organised in New York, St. Petersburg, Florence, Berlin, and Vienna. Henceforward quite as much material is to be found in the Proceedings of these Societies, as in separately published monographs; the central position, effective organisation, and high authority of the French Society making its *Bulletin* especially valuable in this respect.

The special study of the forms of the skull was one of the first departments to become systematised, and has always claimed a predominant voice in the classification of races. Not to recur to Blumenbach, Daubenton, or Camper, the founders of Craniology, Sandifort published the first volume of his "Tabula Craniorum" in 1830; Morton his "Crania Americana" in 1839, and "Crania Ægyptiaca" in 1844, the former especially a model of methodical research. In the following year Retzius communicated to Müller's "Archiv" the importance of the distinction between broad and long skulls; and in 1846 Zeune's "Schädelbildung" proposed a triple classification into high, broad, and long types, corresponding with European, Mongolian, and Negro stems. Ten years later Davis and Thurnam began to publish their great "Crania Britannica"; while in 1857 appeared the admirable "Crania Selecta" of Von Baer. In France, meanwhile, the study has been prosecuted with very great elaboration, and the names of Broca, De Quatrefages, Gratiolet, and Dareste, all founders of the Société d'Anthropologie, and of younger workers like Hamy and Topinard, stand in the first rank in this kind of investigation; the text-books of the two last in particular are invaluable to the student of physical anthropology.

It is not, however, surprising to find that the most conspicuous developments of the study of man in recent years have not been in the purely biological domain. Even the fact of the great advance and complexity of modern civilisation throws back some light upon its origins or modes of growth; the revival of classical scholarship, and the introduction of the comparative method into philology, no less than the extension of the methods and ideas of individual psychology to the treatment of what are well termed the psychical phenomena of corporate life, have contributed new masses of material to our knowledge of what man is; and the idea of criticising and of remodelling our ideas of civil and moral obligation by others which, though different in expression, seem no less binding, seems likely to be fruitful in elucidating the most obscure workings of the human mind. But these are topics which deserve more separate and detailed examination.

